# Optical Properties of a Quantum Dot-Ring System Grown Using Droplet Epitaxy

**DOI:** 10.1186/s11671-016-1518-2

**Published:** 2016-06-24

**Authors:** Gabriel Linares-García, Lilia Meza-Montes, Eric Stinaff, S. M. Alsolamy, M. E. Ware, Y. I. Mazur, Z. M. Wang, Jihoon Lee, G. J. Salamo

**Affiliations:** Instituto de Física, Benemérita Universidad Autónoma de Puebla, Av. San Claudio y, Blvd. 18 Sur Edif. 1IF1, Cd. Universitaria, Puebla, Mexico; Department of Physics & Astronomy, Ohio University, Athens, OH, USA; Institute for Nanoscience and Engineering, University of Arkansas, Fayetteville, AR, USA; College of Electronics and Information, Kwangwoon University, Nowon-gu Seoul, 01897 South Korea

**Keywords:** Quantum dots, Quantum rings, Photoluminescence, Finite element, Lifetime of excitons

## Abstract

Electronic and optical properties of InAs/GaAs nanostructures grown by the droplet epitaxy method are studied. Carrier states were determined by **k·p** theory including effects of strain and In gradient concentration for a model geometry. Wavefunctions are highly localized in the dots. Coulomb and exchange interactions are studied and we found the system is in the strong confinement regime. Microphotoluminescence spectra and lifetimes were calculated and compared with measurements performed on a set of quantum rings in a single sample. Some features of spectra are in good agreement.

**Electronic supplementary material:**

The online version of this article (doi:10.1186/s11671-016-1518-2) contains supplementary material, which is available to authorized users.

## Background

Three-dimensional carrier confinement of self assembled quantum dots (SAQDs) causes discrete energy states which give rise to several properties and applications [1–3]. In particular, for information processing, it is desirable to produce deterministic arrangements of the SAQDs to facilitate selective coupling. The most common method to obtain SAQDs is the Stranski-Krastanow strain-driven growth using molecular beam epitaxy. However, this method has the disadvantages of high densities and random distribution of the dots, making patterned arrangements difficult to achieve and limiting coupling to vertical stacks of SAQDs. An alternative is the droplet epitaxy technique [4, 5]. Using this technique, a variety of new structures have been obtained which show natural formation of two or more quantum dots (QDs) around a quantum ring (QR) structure, providing a potential route to self-assembly of laterally coupled QDs. This new geometry has been intensely studied during the last years [6].

Microphotoluminescence (MPL) in particular has been used to determine carrier and excitonic states of a single nanostructure and compare with theoretical results [7, 8]. Theoretical study of systems such as QRs is challenging due to their large sizes and complex shapes, compared with single QDs. A proper model to describe their properties is then required. Among the theoretical approaches to study electronic properties, the most commonly used are the tight-binding model and **k·p** theory [9]. It is possible to study non-periodic system, such as a single QD, with **k·p** assuming a periodic array of single entities [9].

Carriers in SAQDs are under several intrinsic potentials, such as local strain induced by misfit of lattice constants. This effect has been shown to be very important, particularly for arsenides [10, 11]. Andreev et al. used the continuum approach to calculate strain and piezoelectric potentials in a periodic array of single entities such as QDs of different shapes [12, 13]. On the other hand, it has been reported in experimental results that the distribution of indium in the QDs and QRs is not uniform [14, 15], creating a stronger confinement for electrons at the top of QDs and for holes at the bottom [11, 16].

In the present work, electronic and optical properties of InAs/GaAs nanostructures grown by the droplet epitaxy method are studied. MPL and lifetime measurements were performed on a set of QRs in a single sample. Results are compared with calculations based on **k·p**. method using Andreev’s approach to calculate strain, and In gradient is included. Peak positions of MPL spectra are compared with those calculated with Fermi’s Golden rule (FGR). The exciton lifetime is determined within the oscillator strength framework. Characteristic features of the spectra can be described with our theoretical model.

## Experimental Methodology

### Growth of the Quantum Rings

Samples of QRs were grown by droplet epitaxy with indium flux equivalent to form one monolayer (ML) InAs. A 0.1-ML/s flux was supplied to the GaAs surface to form indium droplets. In order to crystallize the indium droplets into InAs nanocrystals, the sample was kept at 350 °C. The full procedure can be found in [5], whereupon nanostructures with two InAs quantum dots connected by a QR were obtained. With this method, QRs grew randomly distributed, and separation between the tips of the QDs is about 190 nm. The QD heights are about 16.8 ± 0.9 and 18.4 ± 0.7 nm in ultralow density (∼10^6^/cm ^2^) [5]. FWHMs for our sample are 86.6 and 72.7 nm for each QD, respectively. Notice that QDs are slightly different in height. This asymmetry turns out to be very important as will be discussed below.

### Photoluminescence Spectra and Lifetime Measurements

All the spectra were measured in a single-stage spectrometer with a liquid nitrogen cooled CCD, with excitation by a Ti:Sapphire laser tuned to 770 nm and 0.1 *μ*W power in oblique incident. The lifetime measurements were done using time-correlated single photon counting, with the Ti:Sapphire laser producing picosecond pulses and an avalanche photodiode with a timing resolution of 0.5 ns. Finally, obtained data were introduced in the PICO [17] program using an anisotropy reconvolution fitting to obtain the lifetime. In all the measurements, the sample was cooled in an optical cryostat to 7.5 K.

For variable polarization measurements, the orientation of the incident polarization vector was changed with a compensated liquid crystal variable retarder along with a fixed quarter wave retarder between the laser and the sample as follows: the laser light enters in the variable retarder which produces elliptically polarized light, then goes into a quarter wave retarder to become linearly polarized light, but with the polarization vector at a different angle of orientation with respect to the initial laser light. Thus, it is possible to observe how the intensity of the photoluminescence spectrum changes as a function of the orientation of the incident polarization vector.

## Model for Carrier States

### Calculation of Strain

**Fig. 1:**
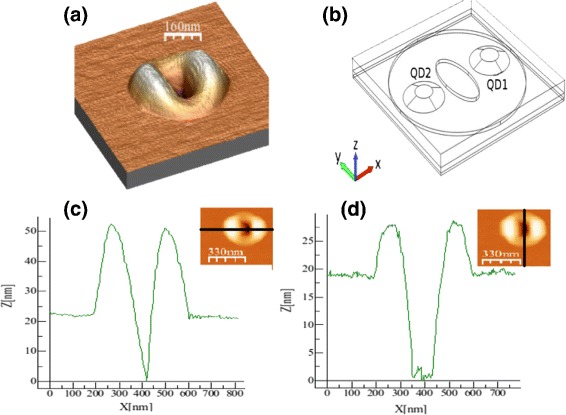
**a** AFM image of a typical QR. **b** Geometrical model of the QR in *xyz* space. **c**, **d** Height of the QR through the directions shown in the insets. The ring radius in the calculation is 336 nm. Notice that the actual box size used in the calculation is larger (900-nm side)

The calculation of the strain tensor *e*_*ij*_ is based on the Fourier transform [12, 13], for a 3D periodic array of QRs in the isotropic case. Sides of the calculation box are large enough compared to the dimensions of the QRs (see Fig. 1). The model fits well because of the ultralow density of QRs.

Sizes and shapes of our QRs in the sample are known by AFM images [5], the shape is important to properly describe symmetry and energy levels [18]. Figure 1 shows the geometry used, where dimensions of the ring are 336 in diameter and 6.5 height. The truncated cone geometry of the dots have radii of 50 on the bases while 23.2 and 22.5 on the top and heights of 10.5 and 12.5 measured from the top of the disc, respectively, as [5] suggested, all dimensions in nm. The big (small) quantum dot is labeled QD2 (QD1) and it is located on the negative (positive) *x* axis. The growth direction is along *z*.

We take spatial averages of the values of strain components. Non-diagonal components of the strain tensor are negligible while the diagonal ones are given in Table 1 for the QR; for the rest of the box calculation, GaAs values were considered [16]. QD1 has the lowest absolute values since it has an smaller amount of InAs, compression being reduced.

**Table 1:** Averaged strain tensor components

*e*_*ij*_	QD1	QD2	Ring
*e*_11_	–0.0348953	–0.0369746	–0.0449516
*e*_22_	–0.0366859	–0.0387921	–0.0470219
*e*_33_	0.0130186	0.0136284	0.0210346

### Gradient of Indium Concentration

Indium concentration *c* depends on geometry, size, nominal composition, and growth method. Determination of the exact profile through the QDs is not simple [15, 19]. Stier et al. [11] have given the variation of the most important parameters of **k·p** method as a function of *c*. We consider the band gap changes in the QR as *E*_*g*_(*z*)(*eV*)=1.518−1.580*c*+0.475*c*^2^.

As an *ansatz*, we chose *c* = 0.56 at the bottom and *c* ≈ 0.59 on the top of QDs, with a linear dependence on *z* [19], the distance in nm measured from the bottom of the ring. This is a reasonable concentration range due to the intermixing between Ga and In which is thought to happen during the ring and nanohole formation [20]. In gradient then follows the relation *c*=0.56+1.5×10^6^*z*. Valence-band deformation potential is *a*_*v*_=−0.220−0.780*c*, and other related parameters were taken from [11, 21]. Dependence on *c* in the Hamiltonian plays a very important role in the carriers states.

### Calculation of Carrier States

Carrier states are computed using a finite-element technique to solve the Schrödinger equation in the framework of 4-band **k·p** theory. The Hamiltonian includes the kinetic energy, geometrical confinement, strain, and In gradient as explained before. Spin degeneracy is ignored. The full expression can be found in [16], successfully applied to arsenide QDs. A small piezoelectric effect is neglected here. Diminishing indium concentration makes the potential increase (decrease) for electrons (holes).

## Results

### Carrier States

**Fig. 2:**
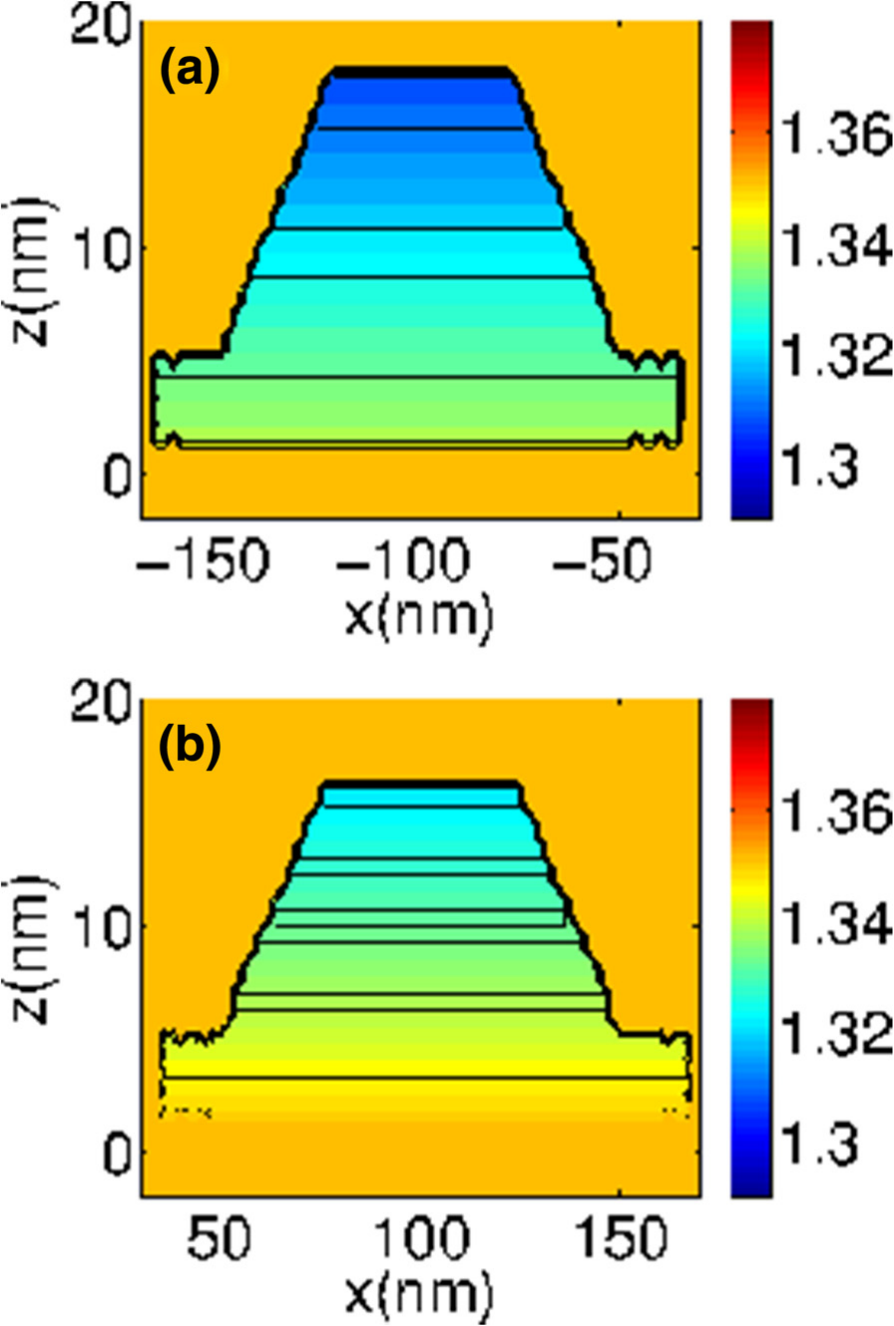
Electron eigenvalues of Hamiltonian at the *Γ* point (*xz* plane), for **a** QD1 and **b** for QD2

Figure 2 shows profiles of the electron eigenvalues of the Hamiltonian at the *Γ* point (**k**=0) in the *xz* plane, with *y* = 0. The spatial-dependent eigenvalues represent the potential field experienced by the carriers. While for electrons, contour levels appear (seen as horizontal lines in the dots) with values decreasing towards the top creating a triangular well potential along *z* direction; this does not happen for holes indicating they are under an uniform potential (not shown here), given by ∼ –0.04(∼ –0.16) eV inside (outside) the QR. The well potential becomes deeper at the top of the dot, causing a strong confinement.

**Fig. 3:**
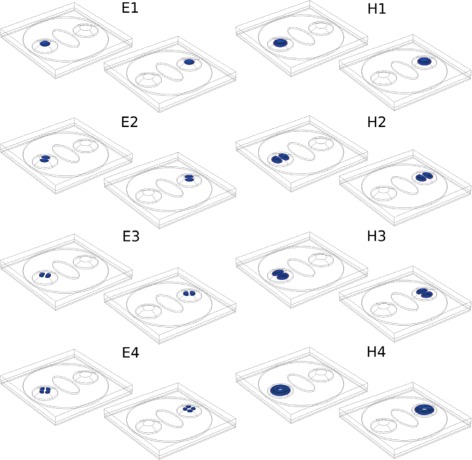
Probability density distribution |*Ψ*(**r**)|^2^ for the four lowest electron (*left*) and holes (*right*) states, calculated in the framework of a 4-band **k** **·** **p** model. *Left columns* show |*Ψ*(**r**)|^2^ for states whose wavefunction is localized in QD2, whereas for *right columns*, it is in QD1. The calculated energies of the electron states are E1 = 1.3185 and 1.3321, E2 = 1.3190 and 1.3325, E3 = 1.3190 and 1.3325, and E4 = 1.3197 and 1.3331 and of the holes are H1 = 0.0125 and 0.0136, H2 = 0.0106 and 0.0117, H3 = 0.0106 and 0.01176, and H4 = 0.0083 and 0.0095, all in eV. Zero energy corresponds to the top of the valence band of bulk InAs

Electron (hole) wave functions for the lowest (highest) values corresponding to the conduction (valence) band are presented in Fig. 3. For a given state, carrier wavefunctions are confined in one dot or the other. Thus, we may refer to the states as if the QDs were isolated, although the wavefunctions were calculated for the whole QR. Electron wavefunctions are localized at the top of the QDs as expected since in that region In concentration is higher and the potential energy lower (see Fig. 2). Hole eigenstates are not very sensitive to the effects of In concentration, and their wavefunctions are distributed through the QD and partially in the ring. It is remarkable that electron energies are determined by the indium gradient and the hydrostatic strain. Equivalent electron states (similar wavefunction but localized at differents dots) do not have the same energy since dots have different sizes as well as there are small changes in the strain distribution (Table 1). For holes, eigenvalues are less separated since they are mainly determined by biaxial strain.

The shape of the carrier wave functions is similar to those of systems with azimuthal symmetry, like a lens [22, 23]. The second and third states have double degeneracy, energies E2 and E3, respectively. Hole wavefunctions behave in the same way. As Table 1 shows, strain is higher in the ring, forcing hole wavefunctions to confine in the dots although they slightly penetrate the ring.

**Fig. 4:**
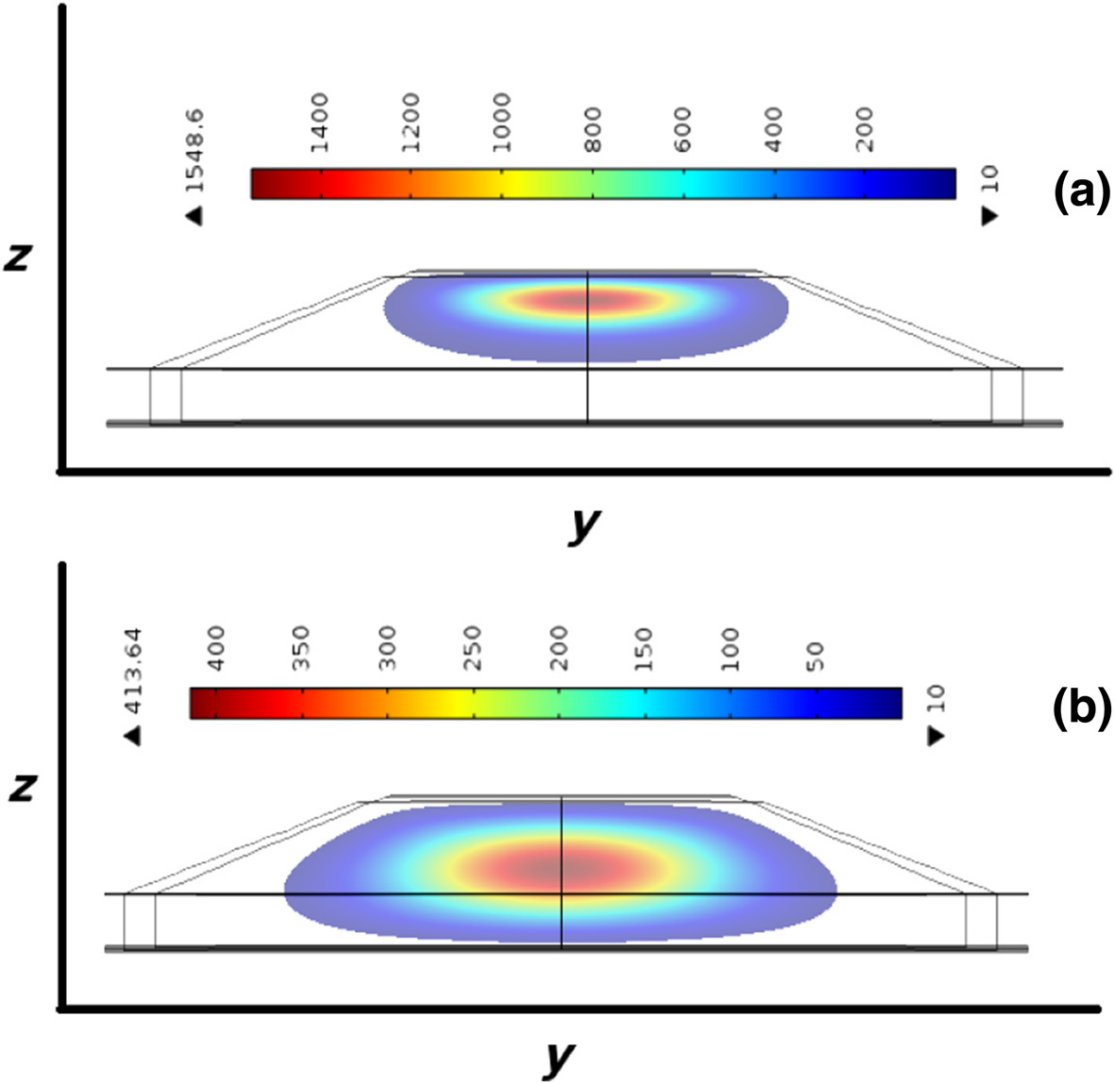
Probability density distribution of carriers in the *yz* plane. **a** Electron E1. **b** Heavy holes H1

Figure 4 shows contour plots of the probability density distribution of (a) electrons and (b) holes of a single QD on the *yz* plane (the behavior in the other QD is similar). Maxima are along the axis of the dots, and the distribution spreads but decays rapidly showing the strong confinement. If these plots were superimposed in a single image (not shown here), we could visualize the overlap between electron and hole wave functions. Here, this overlap is small due to the stronger confinement of the electrons compared to the holes as the figure shows. Nevertheless, it plays a very important role in the MPL spectra and exciton lifetimes.

### Energy binding of neutral excitons

In order to interpret measured emission spectra, we analyze the exciton energy $E_{\text {exc}}=E_{\mathrm {e}}-E_{\mathrm {h}}-J_{\text {ij}}^{\text {eh}}+K_{\text {ij}}^{\text {eh}}\delta _{\text {SO}}$, where (in the absence of spin-orbit coupling) *δ*_SO_=1 for triplet states, and 0 for singlet states. The direct Coulomb *J* describes the electrostatic interaction between electron and hole of energies *E*_e_ and *E*_h_, respectively. Here, we use the dielectric function of bulk InAs for the whole nanostructure since the dielectric constants of InAs and GaAs are similar. The exchange interaction *K* describes the effect of the exchange symmetry of the electron-hole pair [24, 25]. It has been reported that *K* is small compared to *J* [22]. Small overlap of the wavefunctions in the strong confinement regime reinforces this trend so *K* will be neglected. *J* was calculated from the 32 transitions arising of pairs of electron and hole states localized in the same dot. Values of *J* differ within one order of magnitude and their average is 9.5158×10^−5^ (1.0148×10^−4^) eV for states located in QD2 (QD1). These small values are a consequence of the strong confinement. Kinetic-energy effects are then dominant over exchange and correlation further the binding energy decreases as QD volume increases [26]. Thus, emission spectra will be described by means of interband rate transitions (recombination) of unbound electron-hole pairs.

### Theoretical and Experimental Spectra

**Fig. 5:**
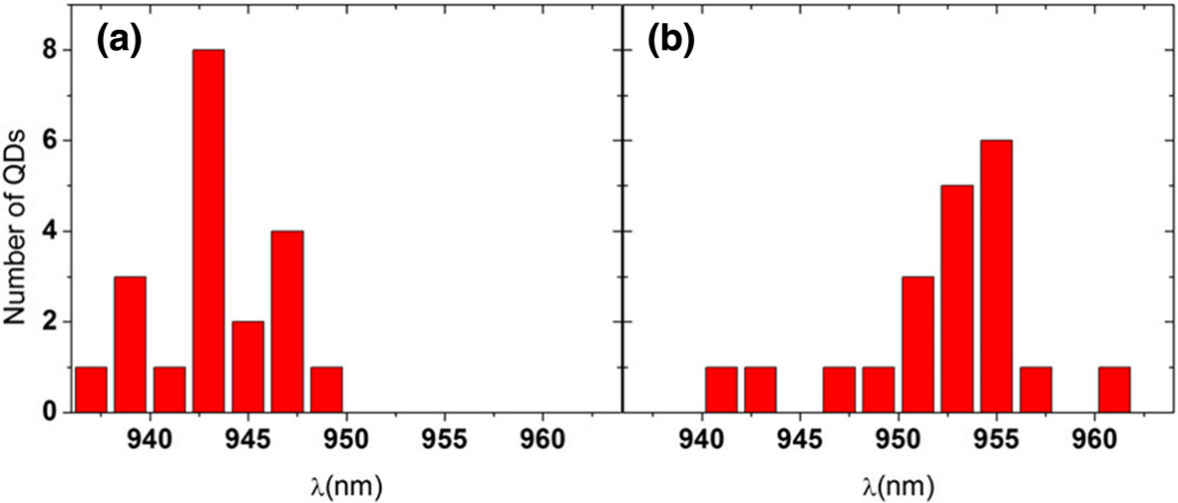
Histogram for positions of the two main peaks of spectra measured for 20 quantum rings of the same sample. **a** Short wavelength. **b** Long wavelength. Average separation between peaks is ≈9 nm

**Fig. 6:**
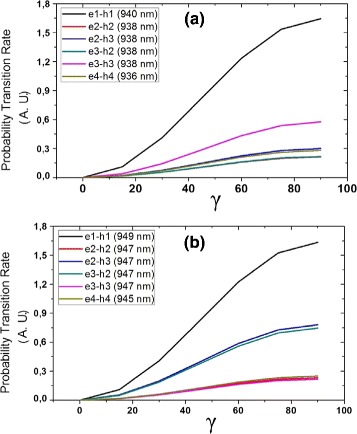
Theoretical probability transition rates for QR model, as a function of the orientation of the vector polarization respect to (–1, 1, 0) for **k** = (1, 1, 1). **a** QD2 (*big*). **b** QD1 (*small*). The corresponding peak position is indicated in nm

Experimental spectra show two main peaks. The peak at short (long) wavelength has been associated with transitions in the small (big) dot [5]. In Fig. 5 histograms of peak positions of 20 QRs indicate that they are centered around 943 and 953 nm, respectively. Since the orientation of QRs is unknown, we can consider that the polarization vector ${\vec {\epsilon }}$ of incident light is randomly oriented (considering oblique incident). Theoretical emission spectra of the QRs were calculated with FGR [27] in the electric dipole approximation. This approximation works at its best in atomic transitions. Since confinement is strong and the excitation wavelength is larger than the dimensions of the system, this approximation is valid in our case. Figure 6 shows the probability transition rates using FGR as a function of the angle between incident linear polarized light and the direction (−1, 1, 0), the latter one chosen arbitrarily as the *γ* = 0°. As an example, wavevector **k** = (1, 1, 1) is assumed. See Additional file 1: Figure S1 for details. The 32 first transitions, 16 for each dot, were calculated; however, only the more intense transitions *e-h* for both quantum dots and they have almost the same intensity due to the similar spatial symmetry of the wave functions (see Fig. 3). For a given ${\vec {\epsilon }}$, two main transitions are observed (one for each dot) of almost the same intensity. They can be assigned to the two main experimental peaks.

**Fig. 7:**
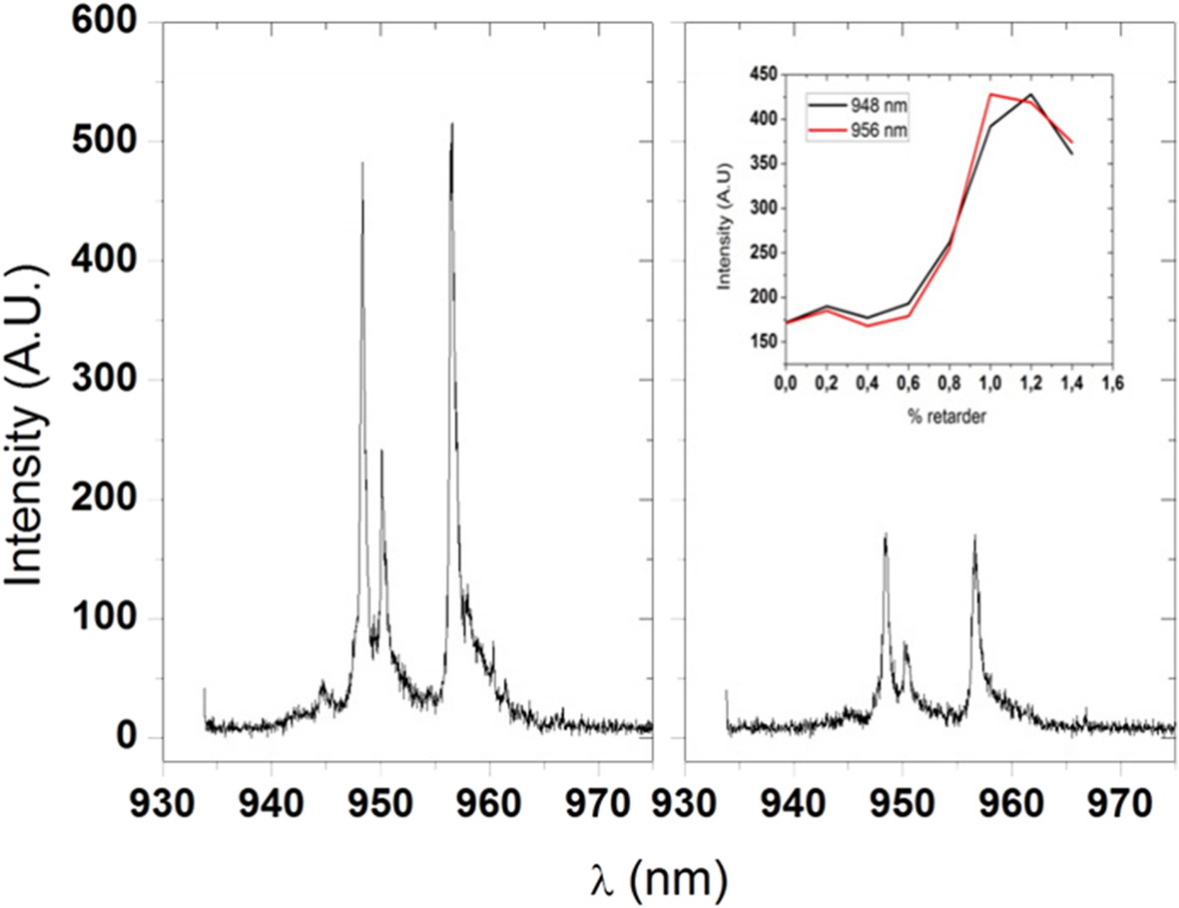
Photoluminescence spectra of a quantum ring, for two different orientations of the vector polarization of the incident light. *Inset*: intensity in the emission as a function of the orientation of vector polarization

In both QDs, the highest probability transition rate is between the states *e*1 and *h*1, having the same value. Others transitions have slight differences since the wave functions of the carriers, mainly the electron, are determined by strain and In gradient which in turn depend on the dot size. Experimentally, we found a small change in the photoluminescence spectra when the polarization vector is rotated as described before (see Fig. 7 for an example). It is not possible to make a quantitative comparison since the actual angles can not be measured. However, the inset shows a similar variation as the theoretical results. On the other hand, we notice that peaks of calculated spectra are slightly blue-shifted with respect to the experimental ones, most likely due to our *ansatz* in QR dimensions and In composition. Nevertheless, the separation between peaks is ∼10 nm, similar to the experimental value as histograms show (Fig. 5).

Experimentally, the separation between peaks is associated with the different sizes of QDs on the QR. In our model, this was included considering different volumes of the QDs, which depend on their heights, i.e., the difference in the heights determines the difference in energy of the spectrum peaks. Thus, in spite of having similar shapes, introducing asymmetry through the QD sizes yields a good agreement with the measured spectral distribution.

### Exciton Lifetimes

**Fig. 8:**
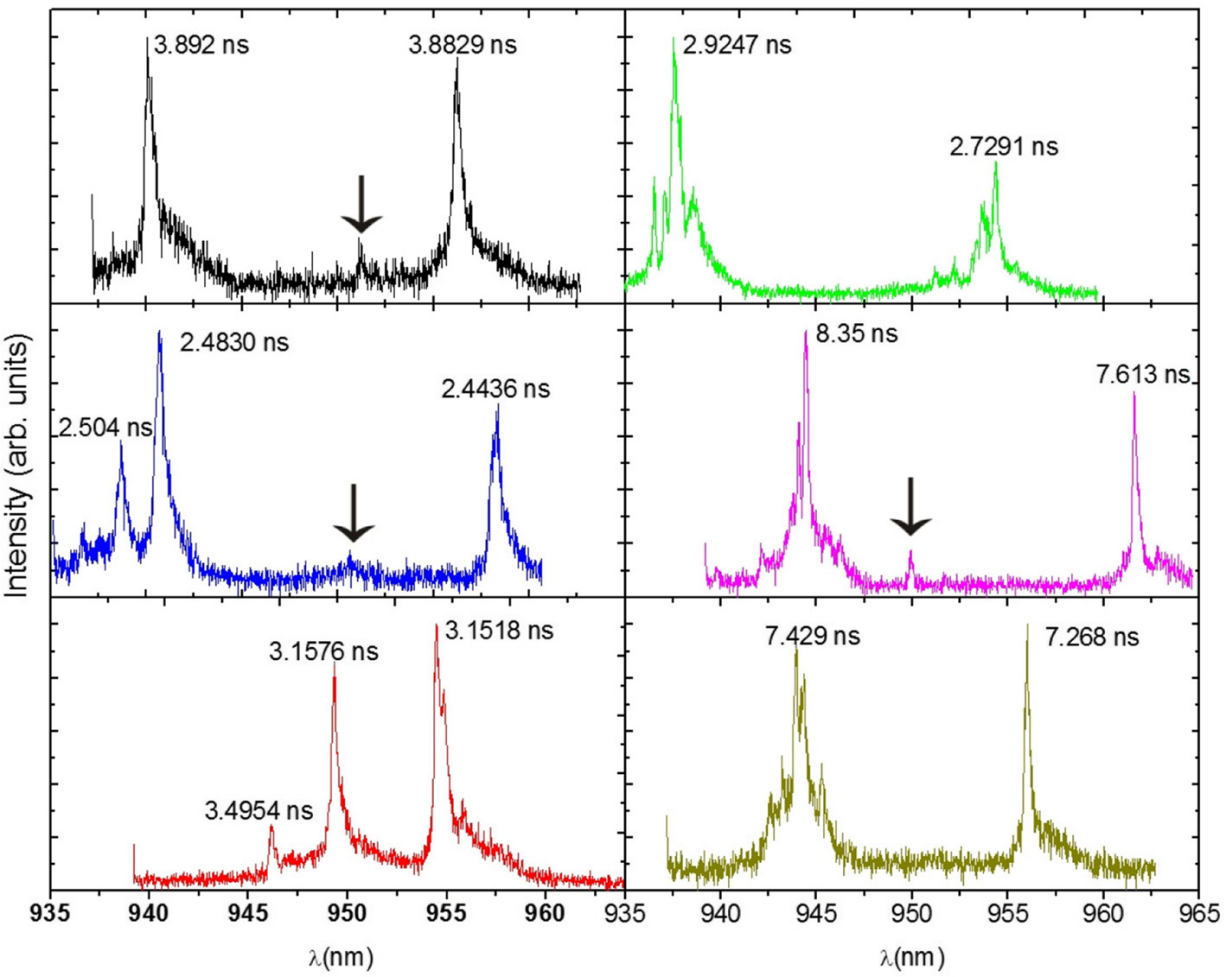
MPL spectra of six QRs measured at 7.5 K. Lifetimes as indicated. The *arrows* near 950 nm indicate satellite peaks

Lifetimes between ≈2 and 8 ns were found for the 18 individual QRs investigated. Figure 8 shows typical QR spectra and their corresponding lifetimes. These spectra were chosen to represent the set because of their shapes.

Within the strong confinement regime, the oscillator strength *f* of the exciton was used to calculated the exciton radiative lifetime [28, 29]. To estimate theoretically the lifetimes we calculate, for the polarization vector $\vec {\mathbf {\epsilon }}$ along the (1,1,1) direction, the lifetime of transition *e1-h1*. We obtained *f* = 5.6593 and 6.8104, *τ* = 0.68 and 0.55 ns for states localized at QD2 and QD1, respectively.

This difference between the theoretical and experimental values of the lifetimes could be related to the lack of accuracy in some quantities like the In concentration and the orientation of the sample as well. Particularly, the In concentration is very important because it strongly determines the strain distribution and therefore the carrier confinement, which in turn changes considerably the overlap and the corresponding oscillator strength.

Notice a satellite peak around 950 nm in some spectra (indicated with an arrow). These satellites appear only for some QRs at variable positions and intensities, as Fig. 8 shows.These peaks could be from charged or multi-exciton peaks, or possibly from excited states of the QDs. Power dependent and photon correlation experiments are currently being performed to investigate these possibilities.

## Conclusions

Low-temperature MPL and lifetimes of neutral excitons have been measured in QRs grown by droplet epitaxy. Additionally, carrier states were calculated within a 4-band **k·p** theory, including strain and In concentration gradient effects in a proposed geometry for the QR. We found that the indium concentration is fundamental to determine the confined electron wavefunctions, while hole states are affected mainly by strain. For the calculated ground and first excited states, wavefunctions are confined within a single dot and we do not find evidence of coupling between quantum dots within our approximation, this probably is due to the large separation between them. From calculations of rate transitions, the two main peaks in MPL spectra can be assigned to transitions between ground electron and heavy hole states. Peak position separations coincide with theoretical calculations, although calculated spectra are slightly blue-shifted. As for the peak intensities, they depend on the orientation of the polarization vector as measurements and calculations show. Calculated lifetimes are within the range of the measured ones. Discrepancies can be attributed to uncertainty in parameters related to dimensions, composition profile and orientation of the QRs. We conclude that our model describes the main characteristics of MPL spectra as well as lifetimes of these complex systems. This contributes to the understanding of these nanostructures.

## Additional file

Additional file 1 Supplementary information. (PDF 83 kb)
