# Supplementary material for: Optical Properties of a Quantum Dot-Ring System Grown Using Droplet Epitaxy
Source: Nanoscale Res Lett. 2016 Jun 24;11:309. doi: 10.1186/s11671-016-1518-2 (PMC4920786; doi:10.1186/s11671-016-1518-2)
Supplement: Additional file 1 — Supplementary information. (PDF 83 kb) [file 11671_2016_1518_MOESM1_ESM.pdf]

Optical properties of a quantum dot-ring system grown using droplet expitaxy

Gabriel Linares-García, Lilia Meza-Montes, Eric Stinaff, S. M. Alsolamy, M. E. Ware, Y. I. Mazur, Z. M.Wang, J. Lee, G.J. Salamo.

Supplementary information

Additional information about the calculations of strain, energy binding of neutral exciton, and exciton lifetime in quantum rings is provided.

Strain tensor. The equation used for the calculation of the strain tensor e_{ij} is

$$e_{ij}(\mathbf{r}) = \frac{(2\pi)^3}{d_1 d_2 d_3} \sum_{n_1,n_2,n_3} \tilde{e}^s_{ij}(\xi_\mathbf{n}) \exp(i\xi_\mathbf{n} \cdot \mathbf{r}), \tag{S.1}$$

where d_1, d_2, and d_3 are the periods of the 3D array of QRs along x, y and z directions, respectively. In the present work, sides of the calculation box $d_1 = d_2 = d_3$ =900 nm are large enough compared to the dimensions of the QRs. Here, $\xi_\mathbf{n} = 2\pi(n_1/d_1, n_2/d_2, n_3/d_3)$ are vectors and $\tilde{e}^s_{ij}$ is the strain tensor in Fourier space. The latter, in the isotropic case, is given by

$$\tilde{e}^s_{ij}(\xi_\mathbf{n}) = \epsilon_0 \tilde{\chi}_{QD}(\xi) \left(\delta_{ij} - \frac{3\lambda + 2\mu}{\lambda + 2\mu} \frac{\xi_i \xi_j}{\xi^2} \right), \tag{S.2}$$

where ϵ_0 is the lattice misfit, $\tilde{\chi}_{QD}$ is the characteristic function, λ and μ are the Lamè constants with values of 0.45×10^{11} Pa and 0.40×10^{11} Pa for InAs, and 0.54×10^{11} Pa and 0.59×10^{11} Pa for GaAs , respectively [12]. The hydrostatic and biaxial strains are defined as $e_h = e_{11} + e_{22} + e_{33}$ and $e_b = 0.5(e_{11} + e_{22}) + e_{33}$, respectively.

Values of the strain are obtained on different planes given by z= 1, 6, 11 and 17 nm, and they are averaged to give values shown in table 1. These values of strain are introduced, together with the parameters of each material and the selected geometry into the Hamiltonian, Ref. [16]. We use the software COMSOL, to get the numerical solution of the eigenvectors (energy of carriers) and eigenfunctions (wave functions) of electrons and holes.

Energy binding of neutral exciton. The wavefunctions (ψ^e_i, ψ^h_i) and energies (E_e, E_h) of carriers are used to get the exciton energy, which is defined as $E_{exc} = E_e - E_h - J^{eh}_{ij} + K^{eh}_{ij}\delta_{SO}$, where the direct Coulomb J is given by

$$J^{eh}_{ij} = \frac{e^2}{4\pi\varepsilon_0\varepsilon_r} \int\int \frac{|\psi^e_i(\mathbf{r}_e)|^2 |\psi^h_j(\mathbf{r}_h)|^2}{|\mathbf{r}_e - \mathbf{r}_h|} d\mathbf{r}_e d\mathbf{r}_h, \tag{S.3}$$

where ε_r is the dielectric function of bulk InAs for the whole nanostructure since the dielectric constants of InAs and GaAs are similar, $\varepsilon_\infty = 12.3$, and 10.6, respectively [22]. Notice that K^{eh}_{ij} is very small so we neglected it.

Exciton Lifetime. Wavefunctions and energies of carriers are used to calculate the exciton lifetime. Within the strong confinement regime, the electron-hole wave function can be written in the Hartree approximation as

$$\psi_{exc}(\mathbf{r}_e, \mathbf{r}_h) = \psi^*_e(\mathbf{r}_e)\psi_h(\mathbf{r}_h). \tag{S.4}$$

The oscillator strength f of the exciton can be calculated as

$$f = \frac{2\hbar^2}{m_0 E_{exc}} \sum_{\alpha} \left| \int \psi_e^*(\mathbf{r})(\epsilon \cdot \mathbf{k})\psi_h^{\alpha}(\mathbf{r})d\mathbf{r} \right|^2, \tag{S.5}$$

where $\mathbf{k} = -i\nabla$ and α denotes different hole wave functions. In low-dimensional structures like QDs, the exciton radiative lifetime [28, 29] is then calculated as

$$\tau = \frac{2\pi\varepsilon_0 m_0 c^3 \hbar^2}{ne^2 E_{exc}^2 f}, \tag{S.6}$$

where $n = 3.5$ is the refraction index of bulk pure InAs.

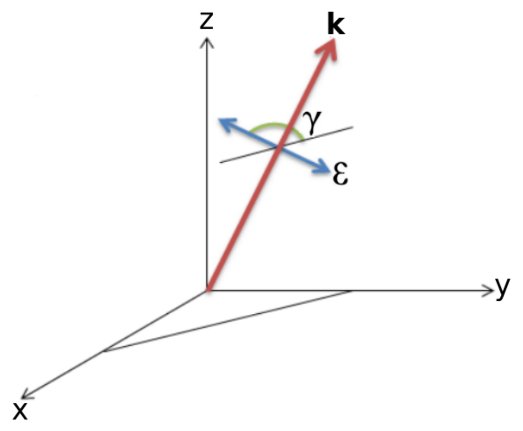

Figure S.1 Scheme showing the orientation of the polarization vector ε of the linearly polarized light. The orientation is given by the angle γ between ε and the direction (-1,1,0), the latter one chosen arbitrarily as the γ=0°. As an example, wavevector $\mathbf{k}$= (1,1,1) is assumed.
